# Preparation and Properties of Low Dielectric Constant Siloxane/Carbosilane Hybrid Benzocyclobutene Resin Composites

**DOI:** 10.3390/ma14216548

**Published:** 2021-11-01

**Authors:** Xian Li, Nan Zhong, Huan Hu, Yufan Zhang, Yawen Huang, Xu Ye, Junxiao Yang

**Affiliations:** 1 School of Materials Science and Engineering, Southwest University of Science and Technology, Mianyang 621010, China; lixian@swust.edu.cn (X.L.); nzhongchem@sina.com (N.Z.); huhuan1112@126.com (H.H.); zyf15181690230@163.com (Y.Z.); 2 State Key Laboratory of Environmental-Friendly Energy Materials, Southwest University of Science and Technology, Mianyang 621010, China; huangyawen@swust.edu.cn (Y.H.); 3 School of Adult and Network Education, Southwest University of Science and Technology, Mianyang 621010, China

**Keywords:** benzocyclobutene resin, BCB-POSS, DVS-BCB, composites, dielectric properties, heat resistance

## Abstract

Benzocyclobutene-modified silsesquioxane (BCB-POSS) and divinyl tetramethyl disiloxane-bisbenzocyclobutene (DVS-BCB) prepolymer were introduced into the containing benzocyclobutene (BCB) unit matrix resin P(4-MB-co-1-MP) polymerized from 1-methyl-1-(4-benzocyclobutenyl) silacyclobutane (4-MSCBBCB) and 1-methyl-1-phenylsilacyclobutane (1-MPSCB), respectively. The low dielectric constant (low-*k*) siloxane/carbosilane hybrid benzocyclobutene resin composites, P(4-MB-co-1-MP)/BCB-POSS and P(4-MB-co-1-MP)/DVS-BCB, were prepared. The curing processes of the composites were assessed via Fourier-transform infrared spectroscopy (FTIR) and differential scanning calorimetry (DSC). The effects on dielectric properties and heat resistance of those composites with different proportion of BCB-POSS and DVS-BCB were investigated using an impedance analyzer and thermogravimetric analyzer (TGA), respectively. The thermal curing of composites could be carried out by ring-opening polymerization (ROP) of the BCB four-member rings of BCB-POSS or DVS-BCB and those of P(4-MB-co-1-MP). With increasing the proportion of BCB-POSS to 30%, the 5% weight loss temperature (*T*_5%_) of P(4-MB-co-1-MP)/BCB-POSS composites was raised visibly, whereas the dielectric constant (*k*) was decreased owing to the introduction of nanopores into POSS. For P(4-MB-co-1-MP)/DVS-BCB composites, the *T*_5%_ and *k* were slightly raised with increasing the proportion of DVS-BCB. The above results indicated that the BCB-POSS showed advantages over conventional fillers to simultaneously improve thermostability and decrease *k*.

## 1. Introduction

With the miniaturization and intelligent development of electronic devices, novel materials with high thermal stability and low-*k* are widely explored [1,2,3,4]. Polymers containing BCB groups can be heated to prepare thermosetting resin via Diels–Alder reaction without adding a catalyst or additives, and no by-products were produced during curing. Therefore, BCB resins have been widely used in the microelectronics industry due to their excellent comprehensive properties [5,6,7,8,9,10]. In order to meet the performance requirements of the next generation of industrial semiconductors, BCB groups have been introduced into linear polymers as thermal cross-linking groups, which can significantly reduce the *k* of the cured resin and enhance the mechanical and thermal properties [11,12,13,14,15,16,17,18]. The introduction of silicone units or other big groups into BCB resins can improve the hygroscopic resistance, thermal stability and dielectric properties of the resin [19,20,21].

With the continuous development of science and technology, the comprehensive performance of dielectric materials (especially those materials used in the fields of aerospace, microelectronics packaging, military, etc.) are required to be more and more excellent. The methods of improving the comprehensive performance of dielectric materials are widely investigated and have been expanded from the traditional molecular structure design to compounds, doping and hybridization [22]. POSS is a highly symmetrical caged structure molecule and could be introduced into the cured resin to prepare hybrid materials, whose special inorganic skeleton (Si‒O‒Si) endows it with excellent thermal stability. Because the caged structure of POSS can provide a large number of air gaps, the dielectric constant of the POSS-hybridized materials can be effectively reduced [23,24,25,26,27,28]. In addition, the eight vertices of POSS can be easily modified to adjust the corresponding properties (thermal stability, mechanical properties, chemical resistance, and dielectric properties, etc.) of the hybrid material through molecular design [29]. Moreover, DVS-BCB resin has the advantages of low-*k*, small dielectric loss, low water absorption and low curing temperature. Meanwhile, DVS-BCB resin has been widely used in microelectronic fields including IC stress buffering/passivation layer, multi-layer wiring, high-frequency devices, and so on [11,30,31].

In this present report, the ROP of 4-MSCBBCB and 1-MPSCB was carried out. Two hybridized resins, P(4-MB-co-1-MP)/BCB-POSS and P(4-MB-co-1-MP)/DVS-BCB, were prepared by the physical blending of copolymer P(4-MB-co-1-MP) with BCB-POSS and DVS-BCB prepolymers, respectively. It is presented that the introduction of BCB-POSS can improve the thermal stability of the cured resins comparing with the matrix materials, which can effectively reduce the *k* of the composite materials. With the addition of DVS-BCB prepolymer, the *k* of the composites was slightly increased, and the thermal stability was improved distinctly. Those siloxane/carbosilane hybrid benzocyclobutene resin composites could be potentially used in microelectronics and other fields.

## 2. Experimental Section

### 2.1. Materials

4-Bromobenzocyclobutene (97%) and DVS-BCB were purchased from Beichuan Ruihui Technology Co., Ltd. (Mianyang, China). BCB-POSS was prepared by our research group; the method can be found in the Supplementary Materials. Methyldichlorosilane (90%), dimethylchlorosilane (98%), octavinyl polyhedral oligomeric silsesquioxane (OVPOSS, AR), chloroplatinic acid hexahydrate (H_2_PtCl_6_·6H_2_O, 95%), tetrahydrofuran (THF, 99.8%, super-dried solvent), and allyl chloride (97%) were purchased from J&K Scientific Ltd. (Beijing, China). Karstedt’s catalyst was purchased from Sam Chemical Technology Co., Ltd. (Shenzhen, China). Magnesium (CP), sodium sulfate anhydrous (CP), 1,2-dibromoethane (CP), petroleum ether (AR), methanol (AR), bromobenzene (AR), toluene (AR), iodine (AR), and other solvents were purchased from Chengdu Kelong Chemical Reagent Co., Ltd. (Chengdu, China). Tetrahydrofuran was dried through JC Meyer solvent drying system (Phoenix SDS, Sacramento, CA, USA); toluene was distilled over sodium-benzophenone before use; other solvents were used without further purification. All manipulation involving air-sensitive materials was carried out in oven-dried glassware under nitrogen.

### 2.2. Characterization

At room temperature, liquid-state ^1^H and ^13^C NMR spectra were obtained with a Bruker Avance-600 spectrometer (Zurich, Switzerland) using deuterated chloroform (CDCl_3_) as the solvent and tetramethylsilane (TMS) as the internal reference. Fourier-transform infrared (FTIR) spectroscopy measurements at 400–4000 cm^−1^ were conducted on a Nicolet iS50 FTIR spectrometer (Thermo Fisher Scientific, Waltham, MA, USA) at room temperature, and the sample films were prepared by casting solutions on potassium bromide (KBr) plates. Both sides of composite resin samples were coated with silver paint to form electrodes, and the capacitance was measured by using a 4294A precision impedance analyzer (Agilent Technologies Co. LTD., Santa Clara, CA, USA) at various frequencies ranging from 40 Hz to 40 MHz at ambient temperature. The dielectric constant (*ε*_r_) was calculated by the following formula $\varepsilon_{r}=(C\times d)/(\varepsilon_{0}\times S)$, where *C*, *d*, and *S* represent capacitance, thickness, and electrode area, respectively. *ε*_0_ represents the permittivity of free space, which is 8.854 × 10^−12^ F/m. Thermogravimetry analysis (TGA) and differential scanning calorimetry (DSC) were performed on a synchronous thermal analyzer (SDT Q600, TA Instruments, New Castle, DE, USA) in nitrogen atmosphere at a heating rate of 10 °C/min.

### 2.3. Preparation of Siloxane/Carbosilane Hybrid Benzocyclobutene Resin Composites

#### 2.3.1. Synthesis of 4-MSCBBCB and 1-MPSCB

All reactions were carried out in a purified nitrogen atmosphere using the standard Schlenk technique. An amount of 15 mL of toluene was injected into a round bottom flask, and a mixture of methyl dichlorosilane (9.00 g, 78.2 mmol), allyl chloride (7.13 g, 93.2 mmol), and chloroplatinic acid (0.4 mL, ~1% THF) was slowly added. The mixture was stirred at 60 °C for 3 h. The excess solvents and raw materials were removed by atmospheric distillation and vacuum distillation successively to obtain 3-chloropropyl-methyl dichlorosilane.

In a nitrogen atmosphere, magnesium ribbons (1.50 g, 62.5 mmol) were put in a three-necked round bottom flask, then THF (5 mL) was added to moisten the magnesium ribbons, and 1,2-dibromoethane (0.2 mL, 2.3 mmol) was added to activate Mg. The mixture of 3-chloropropyl-methyl dichlorosilane (4.98 g, 26 mmol) and THF (10 mL) was added into the reaction system drop by drop, and the reaction mixture was heated to 55 °C and continually stirred for 3 h. After the reaction system cooled down to room temperature, under the protection of nitrogen and with stirring, the mixture of 4-bromobenzocyclobutene (4.76 g, 26 mmol) and THF (18 mL) was added into the reaction system drop by drop. Then the reaction mixture was continually stirred and kept in a reflux state for 2 h. After the reaction was quenched using NaCl solution, the organic layer was extracted by hexane and washed using water, then dried with sodium sulfate anhydrous overnight. The 4-MSCBBCB (3.60 g, 19.1 mmol) was obtained by column chromatography with petroleum ether as eluent, and the yield was 74%. FTIR (KBr plate, cm^−1^): 3055, 2963, 2929, 2858, 1590, 1466, 1395, 1256, 1192, 1119, 860, 720. ^1^H NMR (600 MHz, CDCl_3_) *δ*(ppm): 7.50–7.51 (1H, ArH), 7.36 (s, 1H, ArH), 7.12–7.14 (1H, ArH), 3.22–3.25 (m, 4H, ‒CH_2_CH_2_‒), 2.17–2.22 (m, 2H, ‒C‒CH_2_‒C‒), 1.15–1.33 (m, 4H, ‒CH_2_‒Si‒CH_2_‒), 0.56 (s, 3H, ‒CH_3_). ^13^C NMR (151 MHz, CDCl_3_) *δ*(ppm): 147.62, 145.75, 136.79, 131.78, 127.20, 122.06, 29.93, 29.85, 18.13, 14.55, −1.56.

Replacing 4-bromobenzocyclobutene with bromobenzene, the preparation of 1-MPSCB is similar to the preparation method of 4-MSCBBCB. The yield of it was 76%. FTIR (KBr plate, cm^−1^): 3068, 2963, 2926, 1588, 1396, 1250, 1116, 867, 772, 732, 698. ^1^H NMR (600 MHz, CDCl_3_) *δ*(ppm): 7.41–7.66 (m, 5H, ArH), 2.18–2.24 (m, 2H, ‒C‒CH_2_‒C‒), 1.16–1.33 (m, 4H, ‒CH_2_‒Si‒CH_2_‒), 0.57 (s, 3H, ‒CH_3_). ^13^C NMR (151 MHz, CDCl_3_) *δ*(ppm): 138.68, 133.48, 129.40, 127.91, 18.23, 14.34, −1.79.

#### 2.3.2. Synthesis of P(4-MB-co-1-MP) Copolymer

The polymerization reaction was carried out in a purified nitrogen atmosphere using the standard Schlenk technique. At room temperature, 4-MSCBBCB (1.50 g, 8.0 mmol), 1-MPSCb (1.28 g, 8.0 mmol), and toluene (2 mL) were injected into the anaerobic tube. The anaerobic tube was put into liquid nitrogen to freeze and vacuum for several minutes, then the anaerobic tube was moved out to unfreeze at room temperature under nitrogen conditions. This operation was repeated three times to ensure that the oxygen in the reaction system was removed completely. Then, Karstedt catalyst (5 μL, Pt ~2% xylene) was injected into the reaction system, and the reaction mixture was heated to 70 °C and reacted continuously for 15 h. The colorless viscous liquid was obtained after column chromatography with toluene as eluent, filtration and concentration. P(4-MB-co-1-MP) was recrystallized in methanol 3 times and then dried in a 50 °C vacuum drying oven for 24 h, with a yield of 80%. FTIR (KBr plate, cm^−1^): 3066, 2957, 2920, 2873, 1588, 1457, 1253, 732. ^1^H NMR (600 MHz, CDCl_3_) *δ*(ppm): 7.00–7.38 (ArH), 3.17 (‒CH_2_CH_2_‒), 1.31 (Si‒C‒CH_2_‒), 0.74 (Si‒CH_2_‒C), 0.14 (‒CH_3_). ^13^C NMR (151 MHz, CDCl_3_) *δ*(ppm): 146.61, 145.33, 138.81, 136.80, 133.76, 132.04, 128.60, 127.60, 127.51, 121.76, 29.91, 29.75, 19.16, 18.87, 18.36, −4.73, −5.00.

#### 2.3.3. Preparation of P(4-MB-co-1-MP)/BCB-POSS Composites

BCB-POSS was added to P(4-MB-co-1-MP) at 0 wt.%, 15 wt.% and 30 wt.%, respectively. Then, the mixture was dissolved with toluene and transferred to Durham’s fermentation tube. After solvent volatilization was completed, the Durham’s fermentation tube containing the reaction mixture was placed in a vacuum drying oven and the temperature was elevated and kept at 150 °C for 3 h. After complete degassing, the temperature was kept at 180 °C for 2 h, 200 °C for 2 h, 220 °C for 2 h and 250 °C for 1 h, successively. After curing and cooling to room temperature, the P(4-MB-co-1-MP)/BCB-POSS composite resin sample was obtained.

#### 2.3.4. Preparation of P(4-MB-co-1-MP)/DVS-BCB Composites

In a nitrogen atmosphere, DVS-BCB was heated slowly from room temperature to 175 °C and kept at 175 °C for 3.5 h to obtain DVS-BCB prepolymer. The preparation method of the P(4-MB-co-1-MP)/DVS-BCB composite resin-cured sample from P(4-MB-co-1-MP) and prepolymerized DVS-BCB is similar to that of P(4-MB-co-1-MP)/BCB-POSS.

## 3. Results and Discussion

### 3.1. Preparation and Characterization of 4-MSCBBCB, 1-MPSCB, and P(4-MB-co-1-MP)

**Scheme 1:**
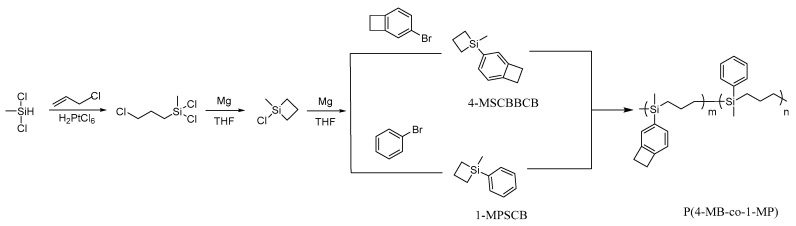
Schematic diagram of synthesis of P(4-MB-co-MP).

In this present work, 4-MSCBBCB, 1-MPSCB, and P(4-MB-co-1-MP) copolymer were prepared and characterized. The schematic diagram of synthesis of P(4-MB-co-MP) is shown in Scheme 1.

The FTIR, ^1^H NMR, and ^13^C NMR spectra of 4-MSCBBCB, 1-MPSCB, and P(4-MB-co-MP) are shown in Figures S1–S4, respectively.

#### 3.1.1. Structure Characterization of 4-MSCBBCB

As the FTIR spectra of 4-MSCBBCB (Figure S1a) show, the peak at 3055 cm^−1^ belongs to the stretching vibration of C‒H bonds of benzene ring, and the stretching vibration peaks of C‒H bonds of methyl and methylene appeared at 2963, 2929, and 2858 cm^−1^, respectively. Moreover, the stretching vibration peaks of C=C bonds of benzene ring appeared at 1590 cm^−1^, the oscillating vibration absorption peaks of the four-member ring of BCB appeared at 1466 cm^−1^, the symmetry deformation vibration peaks at 1395 cm^−1^ and 1256 cm^−1^ belonged to Si‒CH_3_ bonds, the stretching vibration peaks at 1192 cm^−1^ belonged to C‒H bonds of the four-member ring of BCB, the characteristic absorption peaks of silicon heterocyclic butane appeared at 860 cm^−1^ and 1119 cm^−1^, and the planar oscillating vibration absorption peaks of ‒CH_2_‒‒CH_2_‒‒CH_2_‒ appeared at 720 cm^−1^.

As indicated in Figure S2a, for the ^1^H NMR spectra of 4-MSCBBCB, the hydrogen signals at 1.55–2.22 ppm would be assigned to the silicon heterocyclic butane, the hydrogen signals at 3.22–3.25 ppm would be assigned to the four-member ring of BCB, the hydrogen signals at 7.12–7.51 ppm would be assigned to the benzene ring of BCB, and the hydrogen signals at 0.56 ppm would be assigned to the methyl directly attached to the silicon atom.

As Figure S2b (^13^C NMR spectra of 4-MSCBBCB) shows, the characteristic signals of the carbon atoms of benzene ring were found to appear at 147.62–122.06 ppm, the characteristic signals of the carbon atoms of the four-member ring of BCB were found to appear at 29.93 ppm and 29.85 ppm, the carbon signals at 18.13 ppm and 14.55 ppm would be assigned to the methylene of silicon heterocyclic butane. Because of the symmetrical structure, only two sets of signals peaks appeared. Moreover, the carbon signals at –1.56 ppm would be assigned to the carbon atoms on methyl that were directly attached to silicon atom.

#### 3.1.2. Structure Characterization of 1-MPSCB

As the FTIR spectra of 1-MPSCB (Figure S1b) show, the peak at 3068 cm^−1^ belongs to the stretching vibration of C‒H bonds of the phenyl, and the stretching vibration peaks of C‒H bonds of methyl and methylene appeared at 2963 cm^−1^ and 2926 cm^−1^, respectively. Moreover, the stretching vibration peaks of C=C bonds of the phenyl appeared at 1588 cm^−1^, the symmetry deformation vibration peaks at 1396 cm^−1^ and 1250 cm^−1^ belonged to Si‒CH_3_ bonds, the characteristic absorption peaks of silicon heterocyclic butane appeared at 867 cm^−1^ and 1116 cm^−1^, the plane bending vibration peaks at 772 cm^−1^ and 698 cm^−1^ belonged to C‒H bonds of phenyl, and the planar oscillating vibration absorption peaks of ‒CH_2_‒CH_2_‒CH_2_‒ appeared at 732 cm^−1^.

As indicated in Figure S3a (^1^H NMR spectra of 1-MPSCB), the hydrogen signals at 1.16–2.24 ppm would be assigned to the silacyclobutane, the hydrogen signals at 7.41–7.66 ppm would be assigned to the phenyl, and the hydrogen signals at 0.57 ppm would be assigned to the methyl that were directly attached to the silicon atom.

As Figure S3b (^13^C NMR spectra of 1-MPSCB) shows, the carbon signals of the phenyl were found to appear at 138.68–127.91 ppm. The characteristic signals of the carbon atoms of silacyclobutane were found to appear at 18.23 ppm and 14.34 ppm, and because of the symmetrical structure, there were only two sets of signal peaks. Moreover, the carbon signals at –1.79 ppm would be assigned to the methyl that were directly attached to the silicon atom.

#### 3.1.3. Structure Characterization of P(4-MB-co-1-MP)

As Figure S1c shows, for the FTIR spectra of P(4-MB-co-1-MP), the peak at 3066 cm^−1^ belonged to the stretching vibration of C‒H bonds of benzene rings, and the stretching vibration peaks of C‒H bonds of methyl and methylene appeared at 2957, 2920, and 2873 cm^−1^, respectively. The stretching vibration peak of C=C bonds of benzene rings appeared at 1588 cm^−1^. Moreover, the absorption peak of the four-member ring of BCB appeared at 1457 cm^−1^, the symmetry deformation vibration peak at 1253 cm^−1^ belonged to the Si‒CH_3_ bonds, and the planar oscillating vibration absorption peaks of ‒CH_2_‒CH_2_‒CH_2_‒ appeared at 732 cm^−1^. According to the FTIR spectra of P(4-MB-co-1-MP), it was present that the BCB units were successfully introduced into the linear polycarbosilane.

Figure S4a shows the ^1^H NMR spectra of P(4-MB-co-1-MP). Comparing Figure S4a with Figure S2a (^1^H NMR spectra of 4-MSCBBCB monomer) and Figure S3a (^1^H NMR spectra of 1-MPSCB monomer), the hydrogen signals at 1.15–2.22 ppm and 1.16–2.24 ppm disappeared, which would be assigned to the methylenes of silacyclobutane of 4–MSCBBCB and 1-MPSCB, respectively, whereas the new hydrogen signal peaks of 1.31 ppm (Si‒C‒CH_2_) and 0.74 ppm (Si‒CH_2_‒C) in the ^1^H NMR spectra of P(4-MB-co-1-MP) corresponded to the hydrogen of methylene after ring opening. Both sets of signal peaks of the methylene of silacyclobutane of 4-MSCBBCB and 1-MPSCB moved towards the lower chemical shift, indicating that the ROP reactions happened, and the linear polymer was prepared successfully.

The ^13^C NMR spectra of P(4-MB-co-1-MP) are shown in Figure S4b, and the carbon signals of the benzene rings appeared at 146.61–121.76 ppm. The signal peaks of CDCl_3_ were at 77.22, 77.01 and 76.79 ppm. The carbon signal peaks of methylene in BCB appeared at 29.91 ppm and 29.75 ppm, and because of the symmetrical structure, there were only two sets of signal peaks. Moreover, the carbon signal peaks which appeared at 19.16–18.36 ppm corresponded to methylene of the backbone chains of P(4-MB-co-1-MP), and the signal peaks at −4.73 ppm and −5.00 ppm are attributed to the methyl carbon atoms that were directly attached to silicon atoms.

### 3.2. Open-Ring Curing Process of Composite Materials

**Figure 1:**
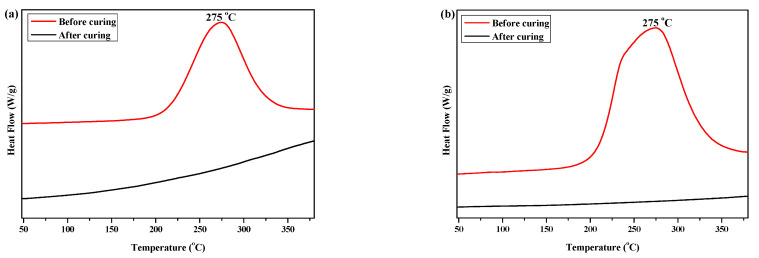

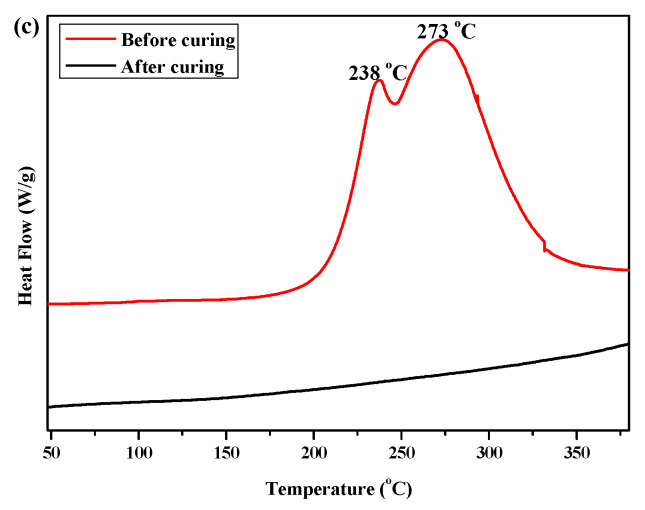
The DSC curves of (**a**) P(4-MB-co-1-MP), (**b**) P(4-MB-co-1-MP)/BCB-POSS, and (**c**) P(4-MB-co-1-MP)/DVS-BCB before and after curing.

DSC and FTIR spectroscopy were used to investigate the curing behaviors of P(4-MB-co-1-MP) and its siloxane/carbosilane hybrid benzocyclobutene resin composites. The DSC curves of P(4-MB-co-1-MP), P(4-MB-co-1-MP)/BCB-POSS, and P(4-MB-co-1-MP)/DVS-BCB (before and after curing) are shown in Figure 1. As Figure 1a shows, the maximum exothermic peak of DSC curves of P(4-MB-co-1-MP) in the nitrogen atmosphere (heating rate of 10 °C/min) appeared at 275 °C, and this peak was attributed to the intramolecular Diels–Alder reaction between *o*-quinodimethanes derived from BCB. However, there were no exothermic peaks in the cured resin DSC curves, indicating that the linear polycarbosilane had been completely cured under the curing conditions shown in the experimental part. Therefore, we suggested that the curing of polycarbosilane was attributed to the ring-opening of the four-member rings of BCB on the side chain to form a highly reactive *o*-dimethylquinone intermediate, which eventually formed cyclooctadiene. It could also be concluded that the thermal cross-linking groups of BCB had been successfully introduced into the P(4-MB-co-1-MP) linear polymers.

**Figure 2:**
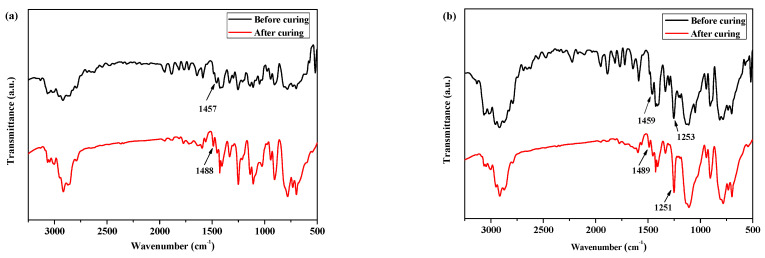

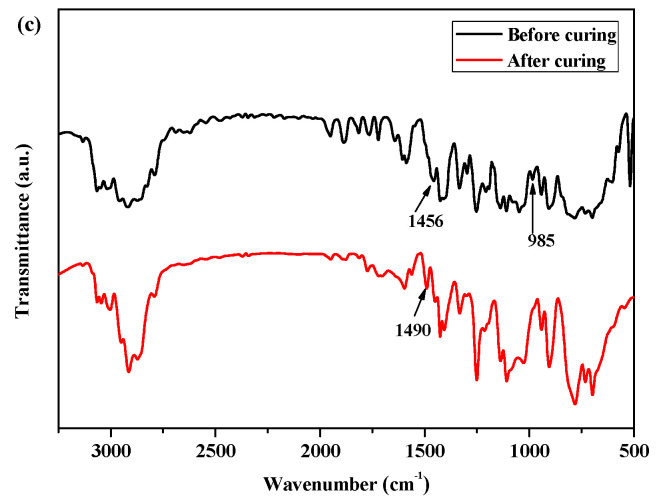
The FTIR spectra of (**a**) P(4-MB-co-1-MP), (**b**) P(4-MB-co-1-MP)/BCB-POSS, and (**c**) P(4-MB-co-1-MP)/DVS-BCB before and after curing.

Moreover, as shown in Figure 2a, for the FTIR spectra of P(4-MB-co-1-MP) before curing, there was a characteristic absorption peak at 1457 cm^−1^, which could be carried out by the four-member ring of BCB. After curing, a new symmetric stretching vibration peak occurred at 1488 cm^−1^, which belonged to the C‒H bonds of methylene obtained from the ring-opening of the four-member ring of BCB.

**Scheme 2:**
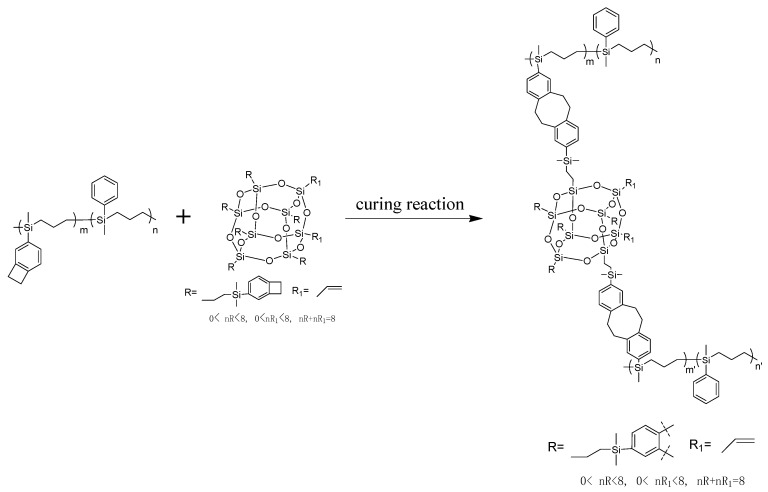
Schematic diagram of the thermal curing mechanism of P(4-MB-co-1-MP)/BCB-POSS.

According to Figure 1b, the maximum exothermic peaks of DSC curves of composites P(4-MB-co-1-MP)/BCB-POSS in nitrogen atmosphere (heating rate of 10 °C/min) appeared at 275 °C, which were caused by the ring-opening of BCB functional groups. After curing, there were no exothermic peaks on the DSC curves of hybrid resin, indicating that it had been cured completely. As shown in Figure 2b, there was a characteristic absorption peak at 1459 cm^−1^ in the P(4-MB-co-1-MP)/BCB-POSS before curing, which belonged to the four-member ring of BCB. After curing, the new C‒H bond symmetric stretching vibration absorption peak of ‒CH_2_‒ due to the ring-opening reaction of BCB appeared at 1489 cm^−1^. The curing mechanism could also be inferred from the FTIR spectra of P(4-MB-co-1-MP)/BCB-POSS before and after curing. Scheme 2 could be the schematic diagram of the thermal curing mechanism of P(4-MB-co-1-MP)/BCB-POSS; the thermal curing process of the composites is depicted visually. During the thermal curing process, the BCB units transferred *o*-dimethylquinone firstly at high temperature, and then the cyclooctadiene was obtained through the Diels–Alder reaction.

**Scheme 3:**
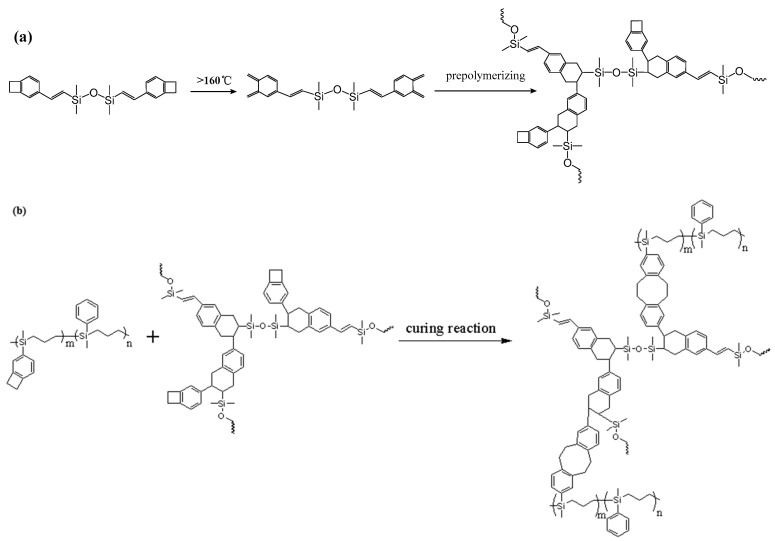
Schematic diagram of (**a**) synthesis of DVS-BCB prepolymer and (**b**) thermal curing mechanism of P(4-MB-co-1-MP)/DVS-BCB.

As shown in Figure 1c, the exothermic peaks of DSC curves of P(4-MB-co-1-MP)/DVS-BCB composites appeared at 238 and 273 °C in the nitrogen atmosphere (heating rate was 10 °C/min), which could be due to the presence of BCB and double bonds in DVS-BCB prepolymers, and the [4 + 2] cycloaddition reaction between double bonds and BCB functional groups occurred during the heating process. The second exothermic peak at 273 °C was due to the formation of cyclooctadiene through the ring-opening addition reaction of the four-member rings of BCB. There was no obvious exothermic peak on the DSC curves of the cured hybrid resin P(4-MB-co-1-MP)/DVS-BCB, which indicated that the curing design could be feasible. Moreover, as shown in Figure 2c, there was a characteristic absorption peak at 1456 cm^−1^ on the FTIR spectra of P(4-MB-co-1-MP)/DVS-BCB before curing, which was due to the four-member rings of BCB. After curing, a new C‒H bond symmetric stretching vibration absorption peak of ‒CH_2_‒, which belonged to the ring-opening reaction of the four-member ring of BCB, appeared at 1490 cm^−1^. As shown in Scheme 3, the linear polycarbosilane was eventually converted into a cross-linking network through the cyclooctadiene structures via the coupling reaction of BCBs.

### 3.3. Thermal Properties of Composites

**Figure 3:**
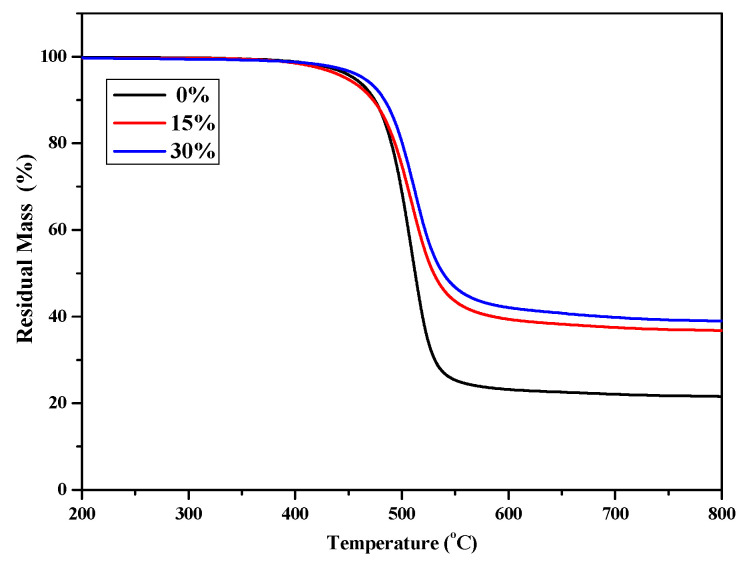
The TGA curves of cured P(4-MB-co-1-MP) and P(4-MB-co-1-MP)/BCB-POSS resins.

TGA was used to evaluate the thermal stability of P(4-MB-co-1-MP), P(4-MB-co-1-MP)/BCB-POSS, and P(4-MB-co-1-MP)/DVS-BCB composites with different proportions of BCB-POSS or DVS-BCB. As shown in Figure 3, the thermal decomposition temperature of weightlessness 5% (*T*_5%_) of P(4-MB-co-1-MP)/BCB-POSS composites was raised to 464.5 °C, when the proportion of BCB-POSS increased to 30%. It was suggested that the cross-linking sites increased with the introduction of BCB of BCB-POSS. In addition, owing to the nanoscale effect, more polymer chains of P(4-MB-co-1-MP) could be located around POSS. As a result, the chain mobility will be decreased. This effect will also contribute to the improvement of thermostability to a certain degree. Therefore, by increasing the proportion of BCB-POSS added, the ratio of carbon residue of P(4-MB-co-1-MP)/BCB-POSS composites increased, and the heat resistance was also improved.

**Figure 4:**
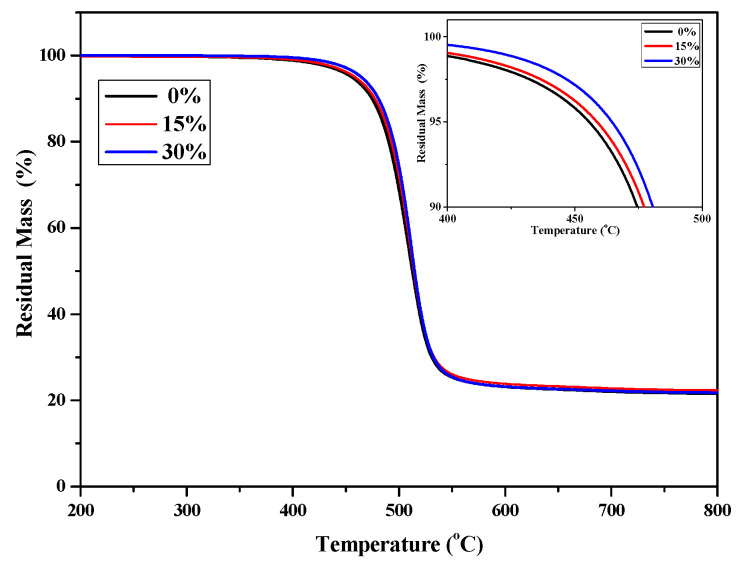
The TG curves of cured P(4-MB-co-1-MP) and P(4-MB-co-1-MP)/DVS-BCB resins.

Figure 4 shows the TGA curves of P(4-MB-co-1-MP) and P(4-MB-co-1-MP)/DVS-BCB with different proportions of DVS-BCB added. *T*_5%_ of P(4-MB-co-1-MP)/DVS-BCB composites also increased with increasing the proportion of DVS-BCB added, and when the proportion of DVS-BCB increased to 30%, *T*_5%_ reached 464.6 °C. It was speculated that the thermal reaction cross-linking sites increased with increasing the proportion of DVS-BCB added, and more stable six-membered ring structures were formed through the addition reaction. Moreover, DVS-BCB contains a certain number of Si‒O bonds with higher bond energy, so the thermal stability of P(4-MB-co-1-MP)/DVS-BCB was better than that of P(4-MB-co-1-MP).

The microelectronics applications of low-*k* materials mainly include inter-layered/lined media and circuit boards. For the former application, the low-*k* materials were required to withstand the thermal anneal from 450 °C under an inert atmosphere. Thus, the *T*_5%_ should be higher than 450 °C. For the latter application, the low-*k* materials were required to be stable at temperatures below 200 °C. Thus, one can conclude that the thermostability of P(4-MB-co-1-MP)/BCB-POSS and P(4-MB-co-1-MP)/DVS-BCB composites satisfies the requirement of microelectronic applications.

### 3.4. Dielectric Properties of Cured Resins

**Figure 5:**
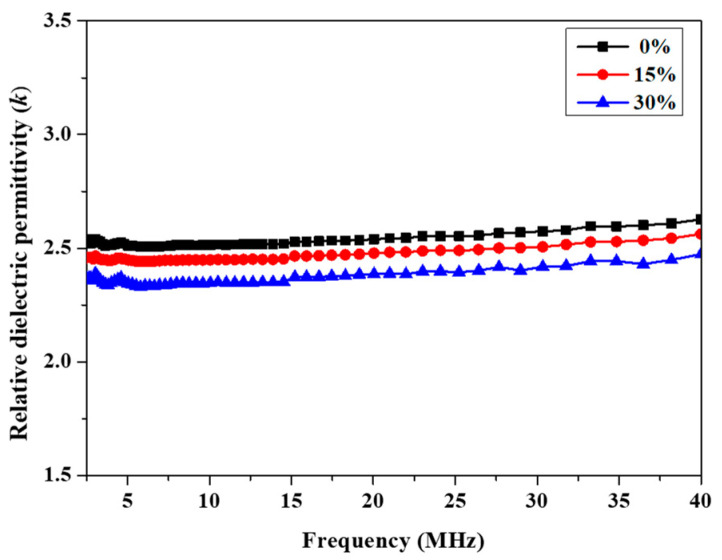
Frequency dependence of relative dielectric permittivity of cured P(4-MB-co-1-MP) and P(4-MB-co-1-MP)/BCB-POSS resins.

**Figure 6:**
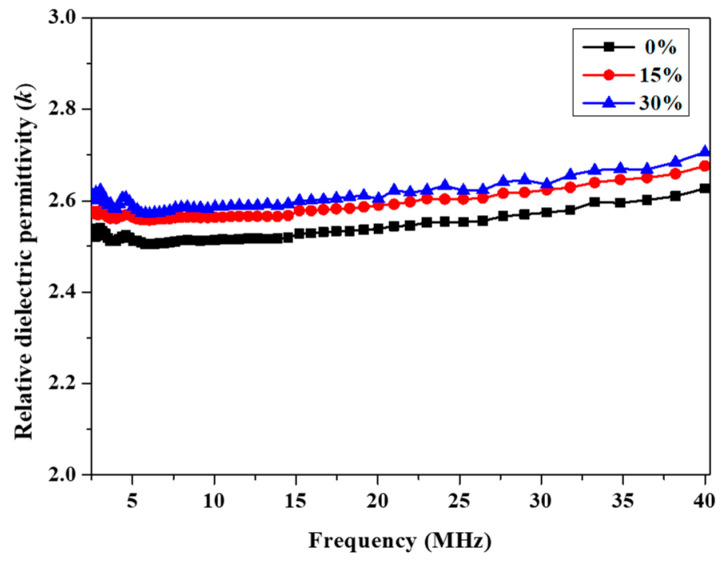
Frequency dependence of relative dielectric permittivity of cured P(4-MB-co-1-MP) and P(4-MB-co-1-MP)/DVS-BCB resins.

The dielectric properties were investigated by means of the impedance method at room temperature. The *k* values of P(4-MB-co-1-MP), P(4-MB-co-1-MP)/BCB-POSS and P(4-MB-co-1-MP)/DVS-BCB cured resins were determined with the frequency range of 40 Hz to 40 MHz. As Figure 5 and Figure 6 show, with the different proportions of BCB-POSS or DVS-BCB in P(4-MB-co-1-MP), the *k* of those cured resins showed a regular trend. According to Figure 5, the *k* of P(4-MB-co-1-MP)/BCB-POSS with different proportions of BCB-POSS were clearly lower than that of P(4-MB-co-1-MP), and the *k* of cured resins decreased with increasing proportions of BCB-POSS added: the *k* of P(4-MB-co-1-MP), P(4-MB-co-1-MP)/BCB-POSS (15 wt.%) and P(4-MB-co-1-MP)/BCB-POSS (30 wt.%) at 10 MHz were 2.52, 2.45 and 2.36, respectively. The *k* of cured P(4-MB-co-1-MP) was 2.52, and there was an obvious decreasing trend after adding BCB-POSS. For example, the dielectric constant (*k*) of P(4-MB-co-1-MP)/BCB-POSS (30 wt.%) decreased from 2.52 to 2.36 (10 MHz) when the proportion of BCB-POSS was 30%. It was indicated that the reasons for the decreases in *k* were mainly due to the special structure similar to holes of POSS, and at the same time, more cross-linking sites brought by BCB-POSS increased the cross-linking density of the composites. Moreover, POSS has a hexahedral hollow structure composed of Si‒O‒Si bonds, and the *k* of air is 1; therefore, the *k* of composites eventually decreased.

As Figure 6 shows, for P(4-MB-co-1-MP)/DVS-BCB, the *k* of the composites increased slightly from 2.52 to 2.65 (10 MHz) with increasing the proportions of DVS-BCB added. It was suggested that although DVS-BCB has two kinds of cross-linking sites, the proportion of Si‒O‒Si bonds in the structure was larger, and the polarizability was higher than those of C‒C bonds and Si‒C bonds, which was an important reason for the increase in *k*.

The results show that there are more advantages of BCB-POSS on enhancing the thermostability and reducing *k* than those of DVS-BCB. The above results indicated that the BCB-POSS showed advantages over conventional fillers to simultaneously improve thermostability and decrease *k*.

## 4. Conclusions

In this work, 4-MSCBBCB and 1-MPSCB were prepared through Grignard reactions, and linear copolymers P(4-MB-co-1-MP) were prepared by Pt-catalyzed ring-opening polymerization. BCB-POSS was prepared by silicon hydrogen addition reaction, and DVS-BCB prepolymer was obtained by prepolymerization. The thermal properties and dielectric properties of cured P(4-MB-co-1-MP)/BCB-POSS and P(4-MB-co-1-MP)/DVS-BCB siloxane/carbosilane hybrid benzocyclobutene resin composites were investigated. The dielectric properties of P(4-MB-co-1-MP)/BCB-POSS composites were improved compared with those of the original matrix resin by adjusting the proportion of BCB-POSS added. The *k* decreased from 2.52 to 2.36 at 10 MHz. At the same time, when the proportion of BCB-POSS reached a certain amount, *T*_5%_ of the composite resins was higher than that of the original matrix resin and the carbon residual ratio was higher, indicating that the thermal stability of the composite resin was improved by hybridizing with inorganic medium. As compared with BCB-POSS, the introduction of DVS-BCB resulted in an increase in the *k* and slight improvement of thermostability. Overall, our work showed that the introduction of POSS-based fillers in P(4-MB-co-1-MP) enabled the decrease in dielectric constant and the enhancement of thermostability owing to its porous and nanoscale effect, and introducing BCB-POSS has more advantages than introducing DVS-BCB.

## Data Availability

Data are contained within the article and Supplementary Materials.

## Supplementary Materials

The following are available online at https://www.mdpi.com/article/10.3390/ma14216548/s1. Section 1: Preparation of modified OVPOSS (BCB-POSS) and Scheme S1. Schematic diagram of synthesis of BCB-POSS. Section 2: FTIR, ^1^H NMR, and ^13^C NMR spectra of partial monomers and polymers, including Figure S1. FTIR spectra of (a) 4-MSCBBCB, (b) 1-MPSCB, and (c) P(4-MB-co-1-MP); Figure S2. (a) ^1^H NMR and (b) ^13^C NMR spectra of 4-MSCBBCB; Figure S3. (a) ^1^H NMR and (b) ^13^C NMR spectra of 1-MPSCB; Figure S4. ^1^H NMR and ^13^C NMR spectra of P(4-MB-co-1-MP); Figure S5. (a) FTIR and (b) ^1^H NMR spectra of BCB-POSS.
