# Supplementary material for: Preparation and Properties of Low Dielectric Constant Siloxane/Carbosilane Hybrid Benzocyclobutene Resin Composites
Source: Materials (Basel). 2021 Nov 1;14(21):6548. doi: 10.3390/ma14216548 (PMC8585461; doi:10.3390/ma14216548)
Supplement: Supplementary file 1 [file materials-14-06548-s001.zip › materials-1392619-supplementary.pdf]

Supplementary Materials

Preparation and Properties of Low Dielectric Constant Siloxane/Carbosilane Hybrid Benzocyclobutene Resin Composites

Xian Li [1,2,†], Nan Zhong [1,2,†], Huan Hu [1,2], Yufan Zhang [1,2], Yawen Huang [2], Xu Ye [1,3,*] and Junxiao Yang [2,*]

[1] School of Materials Science and Engineering, Southwest University of Science and Technology, Mianyang 621010, P. R. China

[2] State Key Laboratory of Environmental-Friendly Energy Materials, Southwest University of Science and Technology, Mianyang 621010, P. R. China

[3] School of Adult and Network Education, Southwest University of Science and Technology, Mianyang 621010, P. R. China

*** Correspondence:**

Xu Ye (yexu@swust.edu.cn); Junxiao Yang (yangjunxiao@swust.edu.cn)

[†] **Authors contributed equally to this work.**

1. Preparation of modified OVPOSS (BCB-POSS)

4-(1',1'-dimethyl) silylbenzocyclobutene (4-DMSHBCB) and BCB-POSS were synthesized through the following literature method reported by our group[1,2]. Magnesium ribbons (0.87 g, 36.3 mmol) and appropriate amount of iodine were added into a 100 mL three-necked round-bottom flask. At room temperature, THF (3 mL) was added to moisten the magnesium ribbons under the condition of nitrogen, and the mixture of 4-bromobenzocyclobutene (5.49 g, 30 mmol) and THF (20 mL) were added drop by drop, the reaction system turned pale gray and black. The reaction mixture was heated to 55 ^{o}C and continued stirring for 2 hours to obtain 4-bromobenzocyclobutene Grignard reagent. After the reaction system cooled down to room temperature, dimethylchlorosilane (2.83 g, 30 mmol, diluted with 24 mL THF) was added using constant pressure drop funnel. Then the coarse 4-DMSHBCB products were obtained by reflux at 65 ^{o}C for 2h. The organic phase was extracted by hexane and washed using water, then dried with sodium sulfate anhydrous overnight. After filtration, the solvent was removed by rotary evaporation, and the colorless oily liquid, 4-DMSHBCB (3.4 g, 24.6 mmol), was obtained by silica gel column chromatography with 82% yield.

Toluene (15 mL), OVPOSS (0.47 g, 0.8 mmol), 4-DMSHBCB (1.22 g, 7.5 mmol), and 8 uL H_2PtCl_6 (0.025 M of THF solution) were injected into a 50 mL round-bottom flask, and the mixture was stirred for 65 h at 80 ^{o}C. The solvent was removed by rotary evaporation, and then the BCB-POSS was precipitated in the iced methanol. FTIR (KBr plate, cm^{-1}): 3066, 2957, 2920, 2873, 1590, 1462, 1256, 1120, 880, 815. ^{1}H NMR (600 MHz, $CDCl_3$) δ(ppm): 7.12-7.45 (m, 3H, ArH), 5.71-6.12 (m, 3H, –CH=CH_2), 3.27 (s, 4H, –CH_2CH_2–), 0.33-0.41 (m, 6H, –CH_3). The FTIR and ^{1}H NMR spectra of BCB-POSS were shown as **Figure S5**.

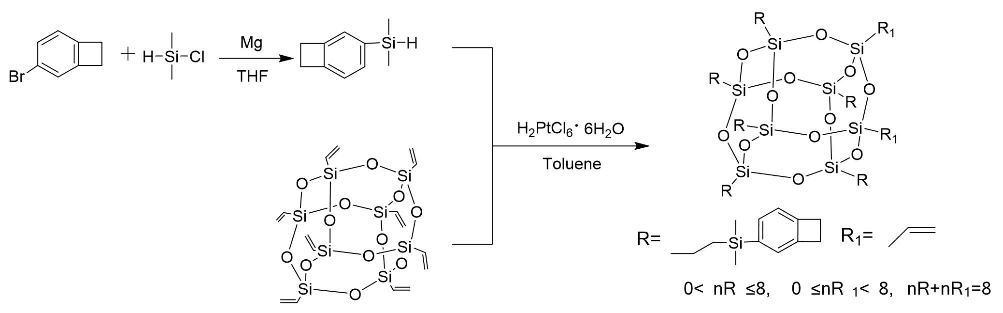

Scheme S1. Schematic diagram of synthesis of BCB-POSS

1. Yang, L.; Cao, K.; Huang, Y.; Chang, G.; Zhu, F.; Yang, J. Synthesis and properties of cross-linkable polysiloxane via incorporating benzocyclobutene. *High Performance Polymers* **2014**, 26, 463–469.
2. Li, S.; Hu, H.; Li, X.; Fan, L.; Wei, X.; Huang, Y.; Yang, J. Preparation and properties of BCB-POSS/benzocyclobutene resin composites. *Polymer Materials Science and Engineering* **2019**, 35, 134–141.

2. FTIR, ^{1}H NMR, and ^{13}C NMR spectra of partial monomers and polymers

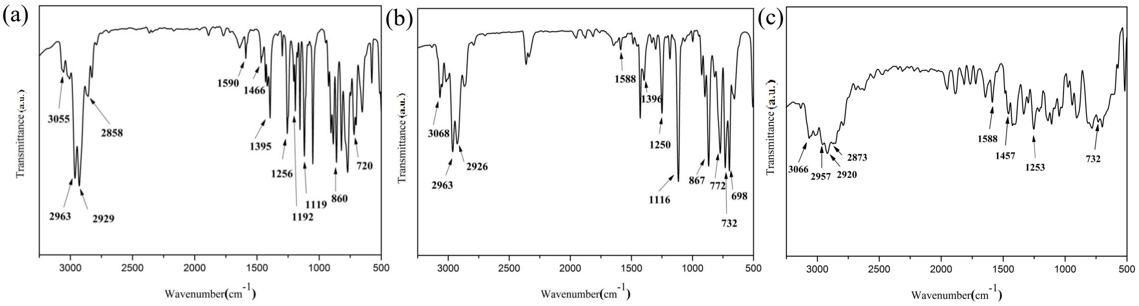

Figure S1. FTIR spectra of (a) 4-MSCBBCB, (b) 1-MPSCB, and (c) P(4-MB-co-1-MP)

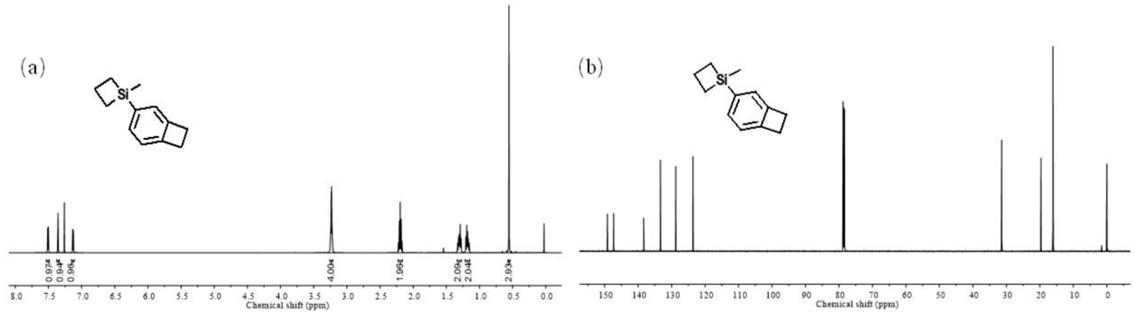

Figure S2. (a) ^{1}H NMR and (b) ^{13}C NMR spectra of 4-MSCBBCB

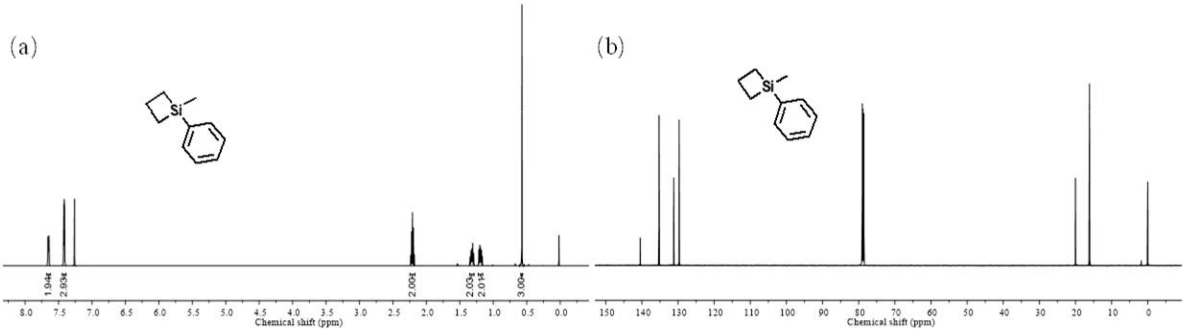

Figure S3. (a) ^{1}H NMR and (b) ^{13}C NMR spectra of 1-MPSCB

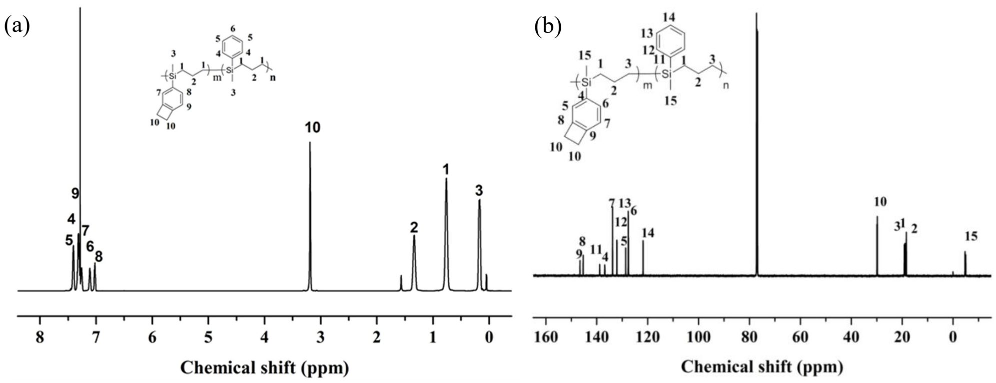

Figure S4. (a) ^{1}H NMR and (b) ^{13}C NMR spectra of P(4-MB-co-1-MP)

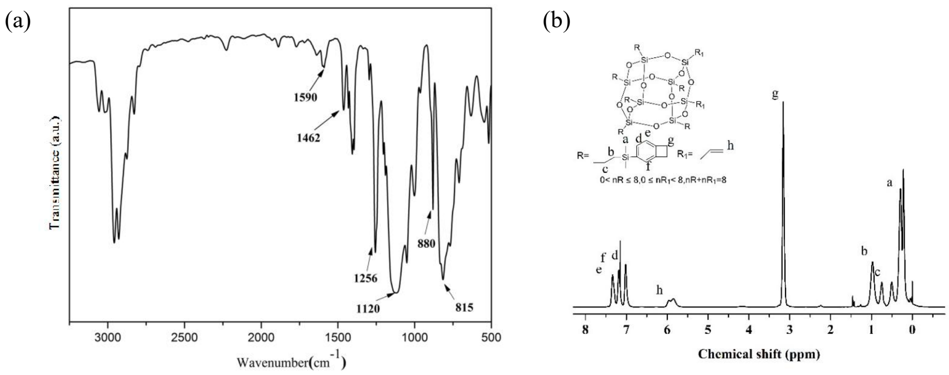

Figure S5. (a) FTIR and (b) ^{1}H NMR spectra of BCB-POSS
